# Targeting nonsense-mediated RNA decay does not increase progranulin levels in the *Grn* ^*R493X*^ mouse model of frontotemporal dementia

**DOI:** 10.1371/journal.pone.0282822

**Published:** 2023-03-09

**Authors:** Denise M. Smith, Michael L. Niehoff, Karen Ling, Paymaan Jafar-Nejad, Frank Rigo, Susan A. Farr, Miles F. Wilkinson, Andrew D. Nguyen

**Affiliations:** 1 Division of Geriatric Medicine, Department of Internal Medicine, Saint Louis University School of Medicine, St. Louis, Missouri, United States of America; 2 Department of Pharmacology and Physiology, Saint Louis University School of Medicine, St. Louis, Missouri, United States of America; 3 Institute for Translational Neuroscience, Saint Louis University, St. Louis, Missouri, United States of America; 4 Veterans Affairs Medical Center, St. Louis, Missouri, United States of America; 5 Ionis Pharmaceuticals, Carlsbad, California, United States of America; 6 Department of Obstetrics, Gynecology, and Reproductive Sciences, University of California San Diego, La Jolla, California, United States of America; 7 Institute of Genomic Medicine, University of California San Diego, La Jolla, California, United States of America; Ohio State University, UNITED STATES

## Abstract

A common cause of frontotemporal dementia (FTD) are nonsense mutations in the progranulin *(GRN)* gene. Because nonsense mutations activate the nonsense-mediated RNA decay (NMD) pathway, we sought to inhibit this RNA turnover pathway as a means to increase progranulin levels. Using a knock-in mouse model harboring a common patient mutation, we tested whether either pharmacological or genetic inhibition of NMD upregulates progranulin in these *Grn*^*R493X*^ mice. We first examined antisense oligonucleotides (ASOs) targeting an exonic region in *Grn*^*R493X*^ mRNA predicted to block its degradation by NMD. As we previously reported, these ASOs effectively increased *Grn*^*R493X*^ mRNA levels in fibroblasts *in vitro*. However, following CNS delivery, we found that none of the 8 ASOs we tested increased *Grn* mRNA levels in the brains of *Grn*^*R493X*^ mice. This result was obtained despite broad ASO distribution in the brain. An ASO targeting a different mRNA was effective when administered in parallel to wild-type mice. As an independent approach to inhibit NMD, we examined the effect of loss of an NMD factor not required for embryonic viability: UPF3b. We found that while *Upf3b* deletion effectively perturbed NMD, it did not increase *Grn* mRNA levels in *Grn*^*+/R493X*^ mouse brains. Together, our results suggest that the NMD-inhibition approaches that we used are likely not viable for increasing progranulin levels in individuals with FTD caused by nonsense *GRN* mutations. Thus, alternative approaches should be pursued.

## Introduction

Progranulin is a lysosomal and secreted protein with pleiotropic effects, including promoting neuronal survival, neurite outgrowth, wound healing, tumor cell growth, and modulating inflammation [1, 2]. In humans, heterozygous *GRN* mutations cause frontotemporal dementia (FTD) due to progranulin haploinsufficiency [3, 4]. Therefore, increasing progranulin levels is a major therapeutic goal [5, 6]. Gene therapy studies in mice provide proof of concept that restoring progranulin levels in heterozygous *Grn* mice improves FTD-associated neuropathology and behavioral deficits [7]. Current therapeutic efforts are focused on small molecules that increase progranulin expression [8–11], gene therapies [7, 12], monoclonal antibodies that modulate progranulin trafficking [13], and protein replacement [14]. However, there are currently no approved therapies for progranulin-deficient FTD.

The vast majority (>80%) of FTD-associated *GRN* mutations are nonsense or frameshift mutations which introduce a premature termination codon (PTC) [15]. As a result, for many of these mutations, the mutant mRNA has been shown to be [3, 4, 16, 17], or is expected to be, degraded through the nonsense-mediated RNA decay (NMD) pathway [18]. Because the progranulin protein contains 7.5 conserved granulin domains, which are believed to be the bioactive units that are produced following proteolytic cleavage, stabilizing mutant *GRN* mRNAs would likely increase the levels of functional granulins. Together, this suggests that inhibiting NMD mechanisms may be feasible therapeutic strategies for increasing levels of progranulin mRNA and functional protein in the context of progranulin-deficient FTD.

NMD can be inhibited by several pharmacological and genetic methods. A number of compounds have been identified which broadly inhibit NMD; these include NMDI1, NMDI9, NMDI14, 5-azacytidine (5AzaC), thapsigargin, and others [19–22]. Another reported strategy for blocking NMD uses antisense oligonucleotides (ASOs), short synthetic oligonucleotides used to modulate target RNAs, to inhibit degradation of a specific PTC-containing transcript [23]. In a cell-based reporter system, Nomakuchi et al. demonstrated that ASOs targeting the exon-junction complex (EJC) at the 3’ end of the exon harboring the PTC can prevent binding of key EJC proteins that are required for NMD, thereby enabling the PTC-containing mutant mRNA to escape NMD-mediated degradation [23]. Most recently, ASO-mediated suppression of the NMD factor UPF3b has been suggested as a potential approach for diseases caused by nonsense mutations [24]. Notably, UPF3b depletion experiments have revealed that UPF3b is a branch-specific NMD factor that regulates a subset of NMD targets [24, 25].

We previously developed a *Grn*^*R493X*^ mouse model that harbors the common *GRN*^*R493X*^ patient nonsense mutation [17], as well as a panel of ASOs that block NMD-mediated degradation of the mutant *Grn*^*R493X*^ mRNA in cultured mouse fibroblast cells [17]. Here, we tested two NMD-targeting strategies for increasing progranulin levels in the brains of *Grn*^*R493X*^ mice. Specifically, we tested the ASOs in a pharmacological approach and *Upf3b* deletion in a genetic approach.

## Materials and methods

### ASOs

ASOs used in these studies were 18-mer ASOs, except the *Malat1*-targeting ASO was a 20-mer. The ASO sequences are provided in S1 Table. ASOs used for *in vitro* and cell-based studies were dissolved in water and stored at -20°C. For *in vivo* studies, lyophilized ASOs were dissolved in sterile PBS without calcium or magnesium (Gibco, 14190–250) and sterilized by passing through a 0.2 μm filter.

### Cell culture

*Grn*^*R493X*^ MEF cells [17] and HeLa cells were cultured in DMEM (Dulbecco’s Modified Eagle Medium, high-glucose) (Gibco, 11995–073) supplemented with 10% fetal bovine serum (FBS) (Gibco, 26140–095), 10 U/ml penicillin, and 10 μg/ml of streptomycin. For ASO treatments, *Grn*^*R493X*^ MEF cells were seeded in 6-well plates, and then transfected as indicated on the following day with 100 nM ASO using 6 μl of Lipofectamine 2000 (Invitrogen). For progranulin expression, HeLa cells were seeded 6-well plates, and then transfected with 1 μg of the indicated plasmid on the following day using 3.75 μl of Lipofectamine 3000 (Invitrogen).

### Mouse studies

Mice were housed in a pathogen-free barrier facility with a 12-h light/12-h dark cycle and provided food and water ad libitum. *Grn*^*R493X*^ knock-in mice [17] and *Upf3b* knockout mice [26] were on the C57BL/6J background and were genotyped by real-time PCR (Transnetyx). For intracerebroventricular (ICV) ASO delivery, 200–500 μg ASO was administered by bolus injection into the right lateral ventricle of mice anesthetized with isoflurane, as previously described [27]. After 2–3 weeks, mice were sacrificed and brain tissues were collected for RNA and protein analyses, as described below. For immunofluorescence, mice were transcardially perfused with PBS followed by 4% paraformaldehyde. For intraperitoneal (IP) ASO delivery, 50 mg/kg of ASO was administered every other day for a total of 4 injections. One day after the final injection, mice were sacrificed and tissues were collected for qPCR analysis.

Animal procedures were approved by the Institutional Animal Care and Use Committee of Saint Louis University (protocol #2764) and followed NIH guidelines. For ICV administration, mice were anesthetized with isoflurane and also provided bupivacaine and buprenorphine. For perfusion, mice were anesthetized with a ketamine/xylazine cocktail followed by transcardial perfusion. For tissue collection, mice were anesthetized with ketamine/xylazine cocktail followed by rapid decapitation.

### RNA analysis

Total RNA was isolated from cultured cells using the RNeasy Mini kit (Qiagen, 74106) with on-column DNase digestion (Qiagen, 79256). RNA was reverse-transcribed to obtain cDNA using the iScript cDNA synthesis kit (Bio-Rad, 1708891), and qPCR was performed using PowerUp SYBR Green Master Mix (ThermoFisher, A25777) with a Bio-Rad CFX384 Real-Time System. Primers sequences are provided in S2 Table. Results for qPCR were normalized to the housekeeping gene 36B4 and evaluated by the comparative C_T_ method.

### Western blot analysis

Mouse cortex samples were lysed in RIPA buffer containing protease inhibitors (Roche, cOmplete Mini EDTA-free Protease Inhibitor Cocktail). Cleared lysates were transferred to new tubes, and protein concentrations were determined using the Bio-Rad DC Protein Assay Kit II. For experiments analyzing secreted progranulin, conditioned media was collected from transfected HeLa cells and cleared at 10,000 x g for 10 min at 4° C. Sample buffer was added to the lysates or conditioned media, and the samples were heated at 95°C for 10 min. Equal amounts of protein lysates (100 μg) or equal volumes of conditioned media (30 μl) were separated on SDS–PAGE gels. Proteins were transferred to nitrocellulose membranes using the Bio-Rad Turbo-Blot transfer system. After blocking and antibody incubations, membranes were incubated with SuperSignal West or SuperSignal Femto chemiluminescent HRP substrate (ThermoFisher) and visualized using a Chemi-Doc system (Bio-Rad). The primary antibodies used for immunoblot analysis include: an anti-mouse progranulin polyclonal antibody (R&D Systems, AF2557, 1:200 dilution) and an anti-α-tubulin monoclonal antibody (Sigma, T9026, 1:2000 dilution). The HRP-conjugated secondary antibodies used were donkey anti-sheep IgG (H+L) (Jackson Immuno Research Labs, 713035147) and donkey anti-mouse IgG (H+L) (Jackson Immuno Research Labs, 715035150).

### Immunofluorescence

Fixed brains were frozen in O.C.T. solution (Tissue-Tek) and sectioned at 40 μm using a cryostat. Free floating sections were blocked and then incubated with a previously described pan-ASO antibody that recognizes the ASO backbone [28] at 1:2000 dilution overnight. After washing, sections were incubated with Alexa Fluor Plus 647 goat anti-rabbit IgG (Invitrogen, A32733, 1:300 dilution) for 1 h, followed by incubation with DAPI (Invitrogen, D1306). After washing, sections were mounted onto slides with Fluoromount-G mounting media (Invitrogen, 00-4958-02). Images were acquired on an Olympus FV1000 confocal microscope with a 20x objective.

### Statistical analyses

Data are presented as means ± SD or means ± SEM, as indicated in the figure legends. Data were analyzed with GraphPad Prism software using the statistical tests described in the figure legends. P values < 0.05 were considered significant.

## Results

We previously developed ASOs that inhibit NMD-mediated degradation of the *Grn*^*R493X*^ mutant mRNA and reported that they increase progranulin mRNA and protein levels in mouse fibroblast cells [17]. These ASOs were designed to block the binding of NMD proteins to the EJC, thereby enabling the *Grn*^*R493X*^ mutant mRNA to escape NMD-mediated degradation. The R493X nonsense mutation is located in exon 12 of the mouse *Grn* mRNA, 159 nucleotides upstream of the next intron; the ASOs target the EJC of exon 12, specifically 17–44 nucleotides upstream of the 3’ end of the exon 12.

**Fig 1:**
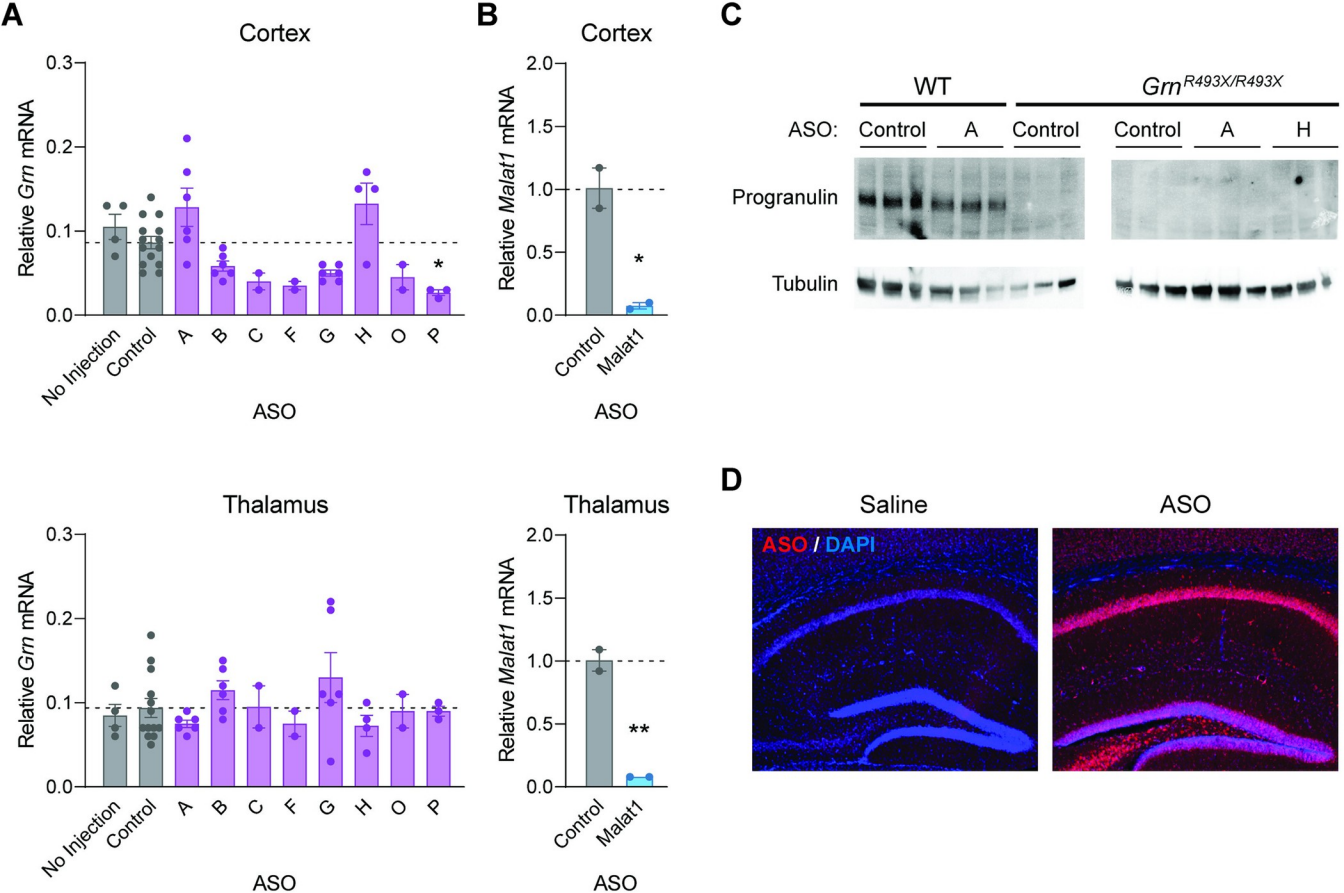
ICV administration of ASOs targeting NMD of the *Grn*^*R493X*^ mRNA does not increase progranulin mRNA or protein levels in the brains of *Grn*^*R493X/R493X*^ mice. qPCR results from brains of *Grn*^*R493X/R493X*^ mice at 2–3 weeks after ICV administration of 200–500 μg ASO. (A) *Grn* mRNA levels are presented relative to levels in tissues of wild-type mice that received control ASO. (B) *Malat1* mRNA levels in wild-type mice. (C) Western blot of mouse progranulin levels in cortex of *Grn*^*R493X/R493X*^ mice at 2 weeks after ICV administration of 500 μg ASO. (D) At 3 weeks after ICV administration of saline or ASO B (200 μg), brains were fixed and sections were stained with an ASO-antibody (red) and counterstained with nuclear stain DAPI (blue). Data are presented as means ± SEM; * indicates p<0.05 and ** indicates p<0.01, as determined by one-way ANOVA with Tukey post hoc test in (A) and by t-test in (B). WT, wild-type.

Here, we report *in vivo* testing of these 8 NMD-targeting *Grn* ASOs in the *Grn*^*R493X*^ knock-in mouse model [17]. In contrast to our findings in cells, we failed to detect any significant increase in *Grn* mRNA levels in the cortex or thalamus of *Grn*^*R493X/R493X*^ mice at 2–3 weeks following ICV administration of 200–500 μg ASO (Fig 1A). Surprisingly, in the cortex, we noted a trend toward decreased *Grn* mRNA levels with multiple ASOs, possibly due to effects on mRNA stability. As a positive control, we administered a validated *Malat1*-targeting ASO that is designed to decrease *Malat1* mRNA levels [29]. As expected, we observed markedly decreased *Malat1* mRNA in the cortex and thalamus (Fig 1B). With the NMD-targeting *Grn* ASOs, we also did not detect any increase in progranulin protein in the cortex (Fig 1C). Importantly, we confirmed that this polyclonal antibody is able to detect the truncated progranulin R493X protein (S1 Fig). Immunofluorescence staining confirmed broad distribution of the ASO throughout the brain in these studies (Fig 1D).

**Fig 2:**
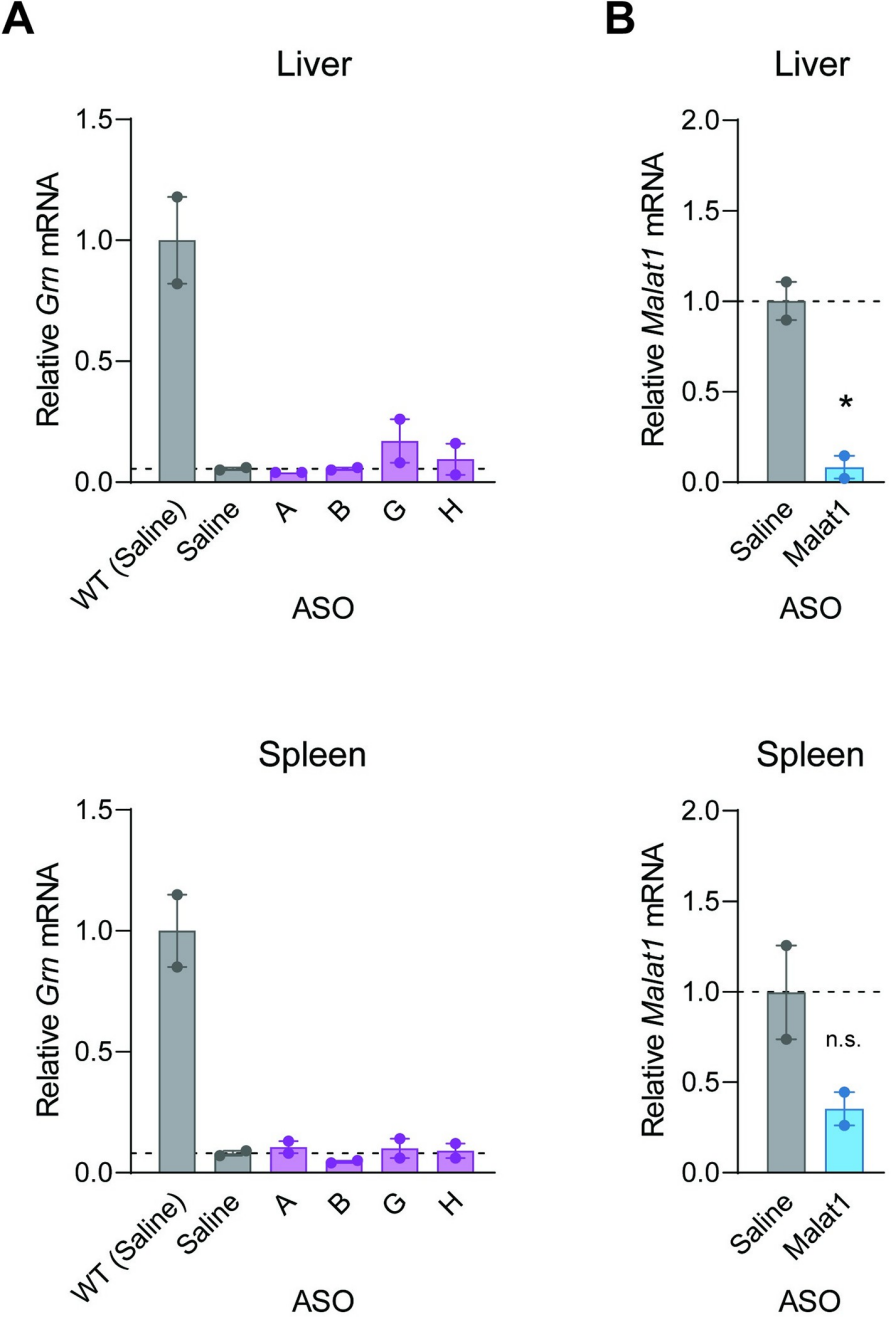
IP administration of ASOs targeting NMD of the *Grn*^*R493X*^ mRNA does not increase *Grn* mRNA levels in the livers and spleens. qPCR results from livers and spleens of *Grn*^*R493X/R493X*^ mice following a series of four IP administrations of 50 mg/kg ASO. (A) *Grn* mRNA levels are presented relative to levels in tissues of wild-type mice that received control ASO. (B) *Malat1* mRNA levels in wild-type mice. Data are presented as means ± SEM; * indicates p<0.05, as determined by one-way ANOVA with Tukey post hoc test in (A) and by t-test in (B). n.s., not significant.

We also performed intraperitoneal (IP) administration and similarly found these ASOs failed to increase *Grn* mRNA levels in the liver and spleen of *Grn*^*R493X/R493X*^ mice (Fig 2A). As a control, the *Malat1*-targeting ASO strongly decreased *Malat1* mRNA in the liver and spleen (Fig 2B). Together, these results demonstrate that the ASOs targeting NMD of the *Grn*^*R493X*^ mRNA failed to increase progranulin levels *in vivo*, despite showing efficacy in cells (S2 Fig) [17].

**Fig 3:**
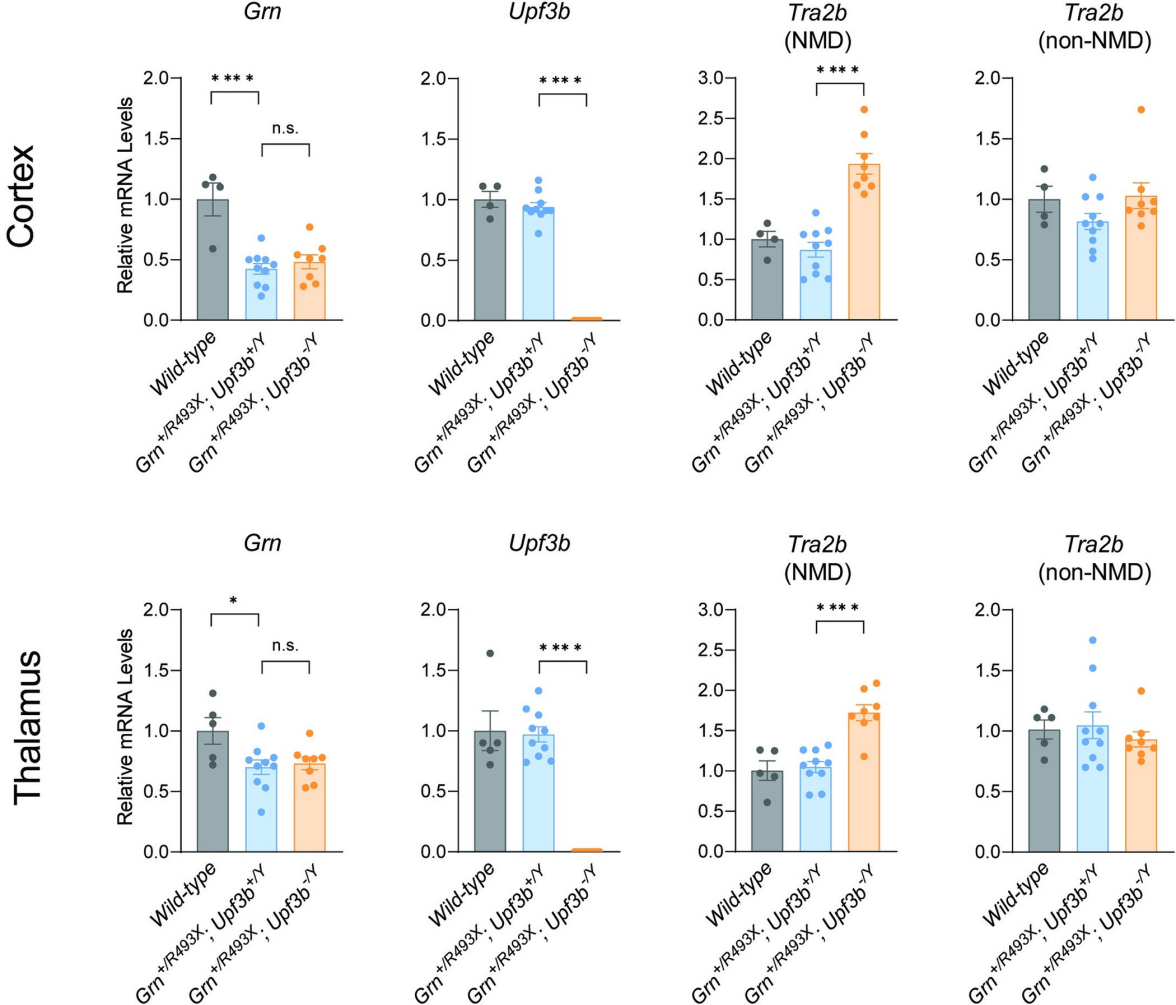
*Upf3b* deletion does not increase *Grn* mRNA levels in the brains of *Grn*^*+/R493X*^ mice. qPCR results from cortex and thalamus of 10–12 week old male mice. *Grn* mRNA levels are presented relative to levels in tissues of wild-type mice. Data are presented as means ± SEM; * indicates p<0.05 and **** indicates p<0.0001, as determined by one-way ANOVA with Tukey post hoc test. n.s., not significant.

There are also recent efforts to block NMD through UPF3b inhibition [24]. Several studies have shown that UPF3b regulates degradation of a subset of NMD transcripts [24, 25]. To determine if the GrnR493X mRNA is regulated in a UPF3b-dependent manner, we crossed GrnR493X mice with Upf3b-null mice [26] and assessed Grn mRNA levels in the brain. Because the Upf3b gene is x-linked, we used UPF3b-expressing male mice (Upf3b+/Y) and UPF3b-deficient male mice (Upf3b–/Y) for comparisons. As expected, Grn+/R493X mice have ~50% Grn mRNA levels compared to wild-type mice, and male Upf3b–/Y mice do not express Upf3b mRNA (Fig 3). In the cortex and thalamus of age-matched mice, Upf3b deletion did not increase Grn mRNA levels; this was in contrast to the established NMD-sensitive isoform of Tra2b mRNA [21], which is significantly increased by UPF3b deficiency. Together, these results suggest that the GrnR493X mRNA is not subject to UPF3b-mediated degradation and therefore not amenable to UPF3b inhibition strategies.

## Discussion

In the current studies, we tested two NMD-targeting strategies for increasing progranulin levels in *Grn*^*R493X*^ mice. In the pharmacological approach, it is unclear why the ASOs targeting NMD-mediated degradation of the *Grn*^*R493X*^ mutant mRNA failed to increase progranulin levels *in vivo*. To rule out delivery issues, we used a validated *Malat1*-targeting ASO as a positive control and observed the expected effect of lowering *Malat1* mRNA levels. We also confirmed broad ASO distribution in the brain by immunostaining. Lastly, after completion of our *in vivo* studies, we confirmed that these ASO stock solutions are inherently active in preventing NMD of the mutant *Grn*^*R493X*^ mRNA when the ASOs were delivered to cultured cells via lipid-based transfection. Together, these results suggest that the NMD-targeting ASOs that are active *in vitro* may not necessarily be active *in vivo*.

The reason(s) for the lack of efficacy of the NMD-targeting ASOs *in vivo* are unclear, but possible reasons include ASO uptake *in vivo* and that the subcellular distribution of these ASOs might not be optimal for them to be efficacious *in vivo*. While there are several reports in cell-based studies [17, 23, 30, 31], to our knowledge there is no demonstration yet of *in vivo* use of ASOs to block NMD by targeting an EJC. Additionally, it is possible that ASO length could be important for targeting NMD *in vivo*; it is worth noting that the NMD-targeting ASOs used in this study are 18-mer ASOs, whereas the positive control *Matat1*-targeting ASO is a 20-mer. This is unlikely to account for the negative results with the NMD-targeting ASOs, because other studies have shown *in vivo* efficacy of centrally administered 18-mer ASOs that sterically block splicing or regulatory factors. For example, an 18-mer ASO targeting an intronic splicing silencer increased the inclusion of exon 7 of *SMN2* in a humanized mouse model of spinal muscular atrophy [32]. Additionally, we recently showed that an 18-mer ASO that blocks a miR binding site in the *GRN* mRNA increases progranulin protein levels in a humanized mouse model [33]. Nonetheless, future studies could test ASOs of different lengths, such as 20-mers, targeting this same EJC region of the *Grn*^*R493X*^ mRNA. Finally, because NMD targeting of an mRNA can vary between cells and tissues [34], we cannot rule out the possibility that the *Grn*^*R493X*^ mRNA is not efficiently targeted to NMD in the brain, despite our previous findings that the mRNA is regulated by NMD in cultured cells and in peripheral tissues [17].

The other major finding, from our genetic approach, is that the *Grn*^*R493X*^ mutant mRNA is not degraded through the UPF3b-dependent branch of NMD. These results further suggest that alternative strategies should be pursued for increasing progranulin levels in the context of progranulin-deficient FTD. One such potential strategy is to use ASOs to block miR binding sites, such as miR-659 and miR-29b [35–38], in the 3’ UTR of the *GRN* mRNA [33]. A notable advantage of this miR-targeting strategy is that it is agnostic to the specific disease mutation and could be used in the context of any of the >70 FTD-associated *GRN* mutations that have been identified [15]. On the other hand, the NMD-targeting strategy would require development of patient-specific ASOs to target the particular exon harboring the nonsense *GRN* mutation.

A limitation of our studies is that they relied heavily on the *Grn*^*R493X*^ knock-in mouse model of progranulin-deficient FTD. While we have previously shown that NMD inhibition similarly increases progranulin mRNA levels in fibroblast cells derived from *Grn*^*R493X*^ knock-in mice and in human fibroblast cells harboring the *GRN*^*R493X*^ mutation [17], we cannot exclude the possibility that species differences may exist with respect to the current findings.

In conclusion, we found that pharmacological inhibition of NMD for the *Grn*^*R493X*^ mRNA and genetic inhibition of the UPF3b-dependent branch of NMD do not increase progranulin levels in the *Grn* ^*R493X*^ mouse model. Our results suggest that these NMD-inhibition approaches are likely not viable for increasing progranulin levels in individuals with FTD caused by nonsense *GRN* mutations. Thus, alternative approaches should be pursued.

## Supporting information

S1 Fig Anti-mouse progranulin antibody detects both wild-type progranulin and R493X truncation mutant. Immunoblot analysis of progranulin using conditioned medium from HeLa cells transfected with plasmids encoding GFP, wild-type (WT) progranulin, or R493X truncation mutant. mPGRN, mouse progranulin.
(PDF)

S2 Fig ASOs targeting NMD of the *Grn*^*R493X*^ mRNA increase *Grn* mRNA levels in *Grn*^*R493X/R493X*^ MEF cells. Cells were transfected with 100 nM ASO using Lipofectamine 2000. After 24 hours, RNA was isolated for qPCR. Grn mRNA levels are presented relative to levels in wild-type cells transfected with control ASO and presented as means ± SD; * indicates p<0.05, ** indicates p<0.01, *** indicates p<0.001, **** indicates p<0.0001, as determined by one-way ANOVA with Dunnett post hoc test.
(PDF)

S1 Table ASO sequences. (PDF)

S2 Table qPCR primer sequences. (PDF)

S1 Raw images Original uncropped western blots used in S1 and S2 Figs. (PDF)
