# Supplementary figures and images for: Targeting nonsense-mediated RNA decay does not increase progranulin levels in the Grn R493X mouse model of frontotemporal dementia
Source: PLoS One. 2023 Mar 9;18(3):e0282822. doi: 10.1371/journal.pone.0282822 (PMC9997918; doi:10.1371/journal.pone.0282822)

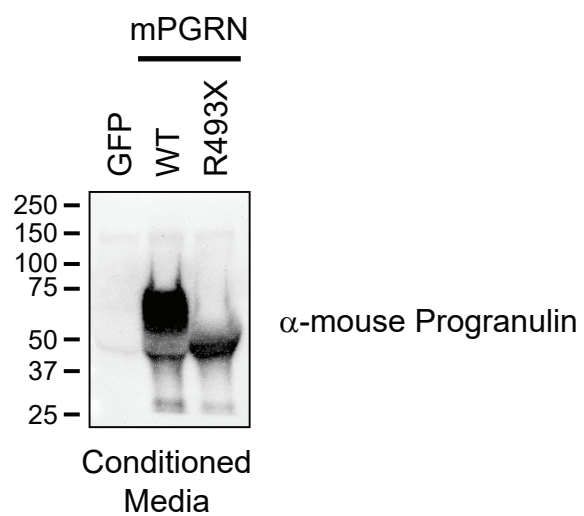

Supplement: S1 Fig — Immunoblot analysis of progranulin using conditioned medium from HeLa cells transfected with plasmids encoding GFP, wild-type (WT) progranulin, or R493X truncation mutant. mPGRN, mouse progranulin. (PDF) [file pone.0282822.s001.pdf]

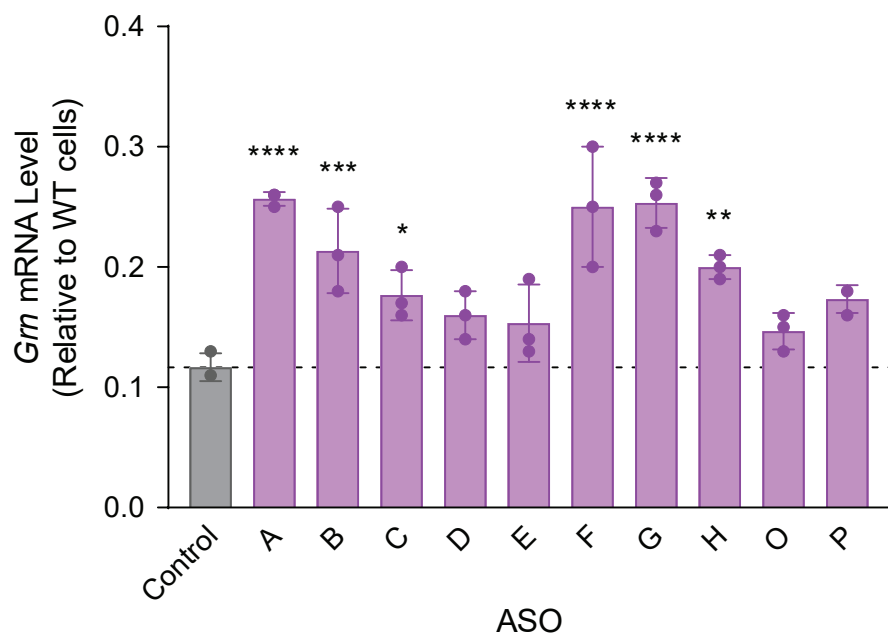

S2 Fig

Supplement: S2 Fig — Cells were transfected with 100 nM ASO using Lipofectamine 2000. After 24 hours, RNA was isolated for qPCR. Grn mRNA levels are presented relative to levels in wild-type cells transfected with control ASO and presented as means ± SD; * indicates p<0.05, ** indicates p<0.01, *** indicates p<0.001, **** indicates p<0.0001, as determined by one-way ANOVA with Dunnett post hoc test. (PDF) [file pone.0282822.s002.pdf]

Figure 1C

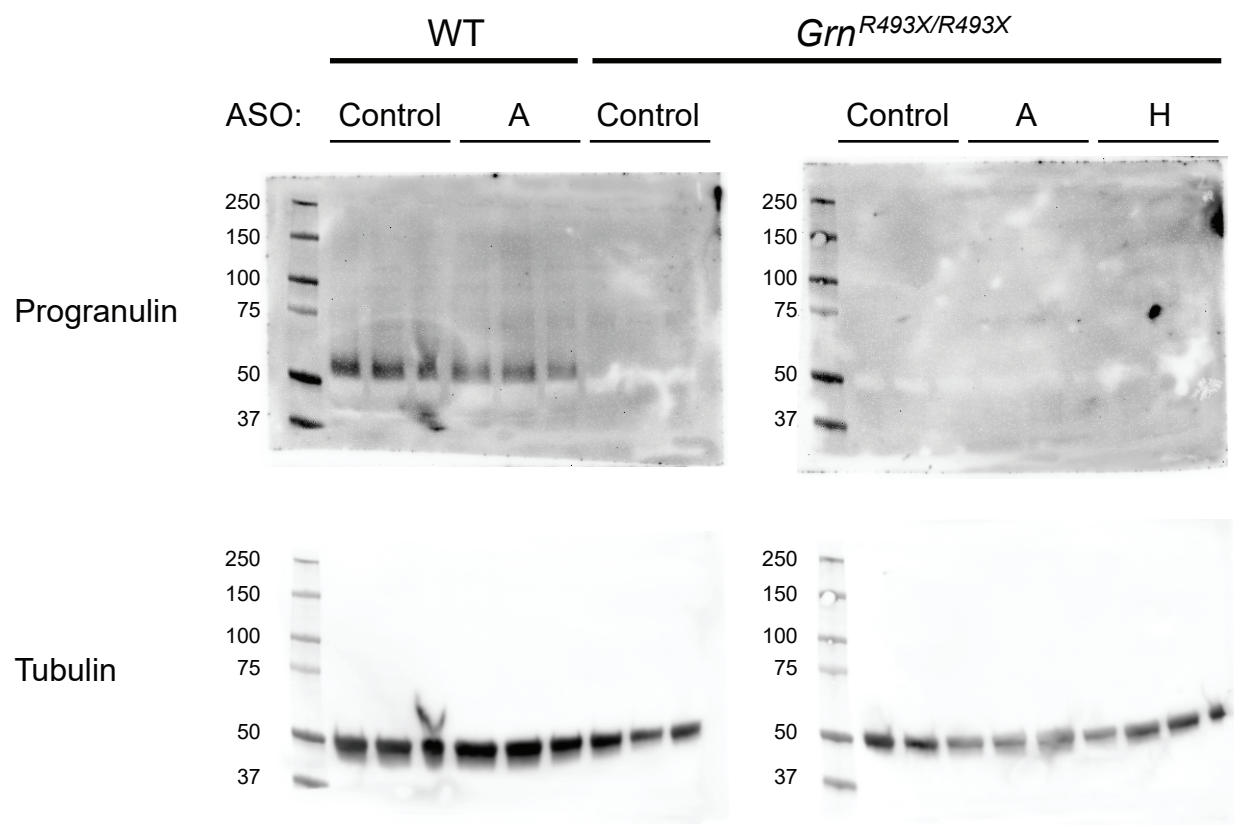

Figure S1

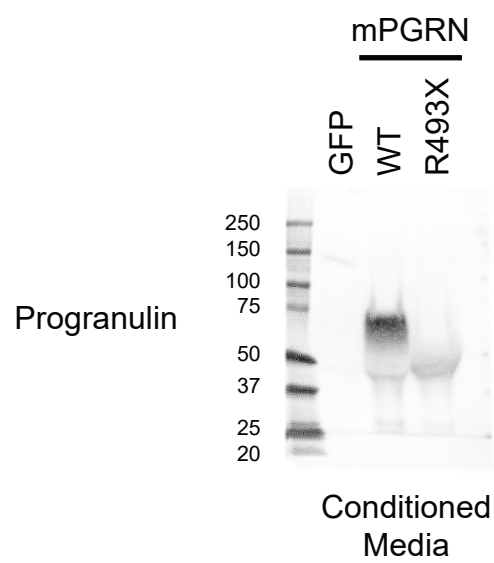

Supplement: S1 Raw images — (PDF) [file pone.0282822.s005.pdf]
