# Supplementary material for: Targeting nonsense-mediated RNA decay does not increase progranulin levels in the Grn R493X mouse model of frontotemporal dementia
Source: PLoS One. 2023 Mar 9;18(3):e0282822. doi: 10.1371/journal.pone.0282822 (PMC9997918; doi:10.1371/journal.pone.0282822)
Supplement: S1 Table — (PDF) [file pone.0282822.s003.pdf]

S1 Table. ASO sequences.

ASO	Sequence
Control	TTAGTTTAATCACGCTCG
A	CTCCTGCACTGTCTTTAC
B	ACTCCTGCACTGTCTTTA
C	GACTCCTGCACTGTCTTT
D	CAGACTCCTGCACTGTCT
E	CCAGACTCCTGCACTGTC
F	GCCCAGACTCCTGCACTG
G	GGCCCAGACTCCTGCACT
H	AGGCCCAGACTCCTGCAC
O	AGACTCCTGCACTGTCTT
P	CCCAGACTCCTGCACTGT
Malat1	GCCAGGCTGGTTATGACTCA
