# Supplementary material for: Targeting nonsense-mediated RNA decay does not increase progranulin levels in the Grn R493X mouse model of frontotemporal dementia
Source: PLoS One. 2023 Mar 9;18(3):e0282822. doi: 10.1371/journal.pone.0282822 (PMC9997918; doi:10.1371/journal.pone.0282822)
Supplement: S2 Table — (PDF) [file pone.0282822.s004.pdf]

S2 Table. qPCR primer sequences.

**Gene**	**Primer sequence**
36B4	Fwd: CACTGGTCTAGGACCCGAGAAG<br>Rev: GGTGCCTCTGAAGATTTTCG
Grn	Fwd: TGGTTCACACACGATGCGTTTCAC<br>Rev: AAAGGCAAAGACACTGCCCTGTTG
Malat1	Fwd: AACCAGTTTCCCCAGCTTTT<br>Rev: CTACATTCCCACCCAGCACT
Tra2b<br>(NMD-sensitive isoform)	Fwd: TGGAATCAGAAAGCACTACGC<br>Rev: GAGTCTTCCTTGGAGCGAGA
Tra2b<br>(NMD-insensitive isoform)	Fwd: GGAGCTTGACAGCTTCAGGA<br>Rev: AAGCAGAACGGGATTCCC

Fwd, forward; Rev, reverse.
